# Genetic Testing in Gastrointestinal Polyposis Syndromes: Considerations in Pediatrics

**DOI:** 10.3390/genes17060601

**Published:** 2026-05-24

**Authors:** Suzanne P. MacFarland, Kristin Zelley, Isabel Rojas, Carol Durno

**Affiliations:** 1 Division of Oncology, Children’s Hospital of Philadelphia, Philadelphia, PA 19104, USA; 2 Division of Pediatrics, University of Pennsylvania Perelman School of Medicine, Philadelphia, PA 19104, USA; 3 University of Texas Southwestern Medical Center, Dallas, TX 75390, USA; 4 Hospital for Sick Children, Toronto, ON M5G 1E8, Canada; 5 The Zane Cohen Cetner, Mount Sinai Hospital, The University of Toronto, Toronto, ON M5G 1X5, Canada

**Keywords:** juvenile polyposis syndrome, Peutz-Jeghers syndrome, PTEN hamartoma tumor syndrome, familial adenomatous polyposis, polyposis syndromes

## Abstract

Pediatric gastrointestinal polyps are frequently associated with an underlying hereditary syndrome associated with multisystem manifestations and increased risk of early-onset cancer. Thus, the identification of polyps in a child should prompt evaluation with genetic testing to (1) characterize the syndrome to determine next clinical steps including surveillance recommendations, and (2) conduct cascade testing to identify affected family members. Given the considerations for pediatric genetic testing, including autonomy and psychosocial stressors associated with the early detection of a cancer risk syndrome, it is important to conduct targeted testing. Herein, we propose a stepwise approach to genetic testing in the pediatric patient with gastrointestinal polyps.

## 1. Introduction

The identification of gastrointestinal (GI) polyps in a pediatric patient requires thoughtful clinical follow-up, often with the implication of lifetime cancer screenings for the individual. Prognosis, the need for additional clinical management, and lifelong cancer risk are highly dependent upon both the clinical context and the underlying genetics. For this reason, genetic testing should be considered in most pediatric patients in whom polyps are identified. However, given the specific considerations for genetic testing in pediatric patients, a systematic approach to this testing is crucial.

Herein, we outline a proposed algorithm to approach genetic testing in pediatric polyposis syndromes. This is divided into two major histologic categories, hamartomas and adenomas, and will also address rare polyp types. Importantly, an accurate pathologic diagnosis is key to the recognition of specific clinical syndromes and selection of the appropriate test. We also discuss considerations for single-gene versus larger panel testing in pediatric patients. This framework is based on a targeted review of the literature, current society guidelines, and expert clinical experience, with the goal of providing a practical and reproducible approach for clinicians.

## 2. Methods

This manuscript was developed as a narrative review with the aim of proposing a practical framework for genetic testing in pediatric patients with gastrointestinal polyps. A targeted literature review was performed using PubMed to identify the relevant studies published in English, focusing on pediatric polyposis syndromes, hereditary gastrointestinal cancer predisposition, and genetic testing strategies. Key search terms included “pediatric polyposis,” “juvenile polyposis syndrome,” “familial adenomatous polyposis,” “Peutz-Jeghers syndrome,” “PTEN hamartoma tumor syndrome,” and “genetic testing in children.”

In addition to the primary literature, current clinical practice guidelines and consensus statements from major societies, including the American Association for Cancer Research (AACR), the North American/European Societies for Pediatric Gastroenterology, Hepatology, and Nutrition, (NASPGHAN/ESPHGAN) and the National Comprehensive Cancer Network (NCCN), and others were reviewed. Sources were selected based on relevance to pediatric populations and clinical applicability.

The proposed algorithms were developed through a synthesis of the available evidence, existing guideline recommendations, and the authors’ clinical experience in pediatric gastroenterology and hereditary cancer predisposition. Where evidence-based guidelines exist, these are reflected in the recommendations, and thus the methods consisted only of summarizing existing and established guidance. In areas where pediatric-specific data are limited or absent, recommendations are based on published data and expert opinion, with an emphasis on balancing diagnostic yield, clinical impact, and the unique ethical considerations of genetic testing in children. In cases where consensus guidelines are not available, lack of data are clearly articulated. This includes consideration of testing implications in children and adolescents.

## 3. Hamartomatous Polyps

**Figure 1:**
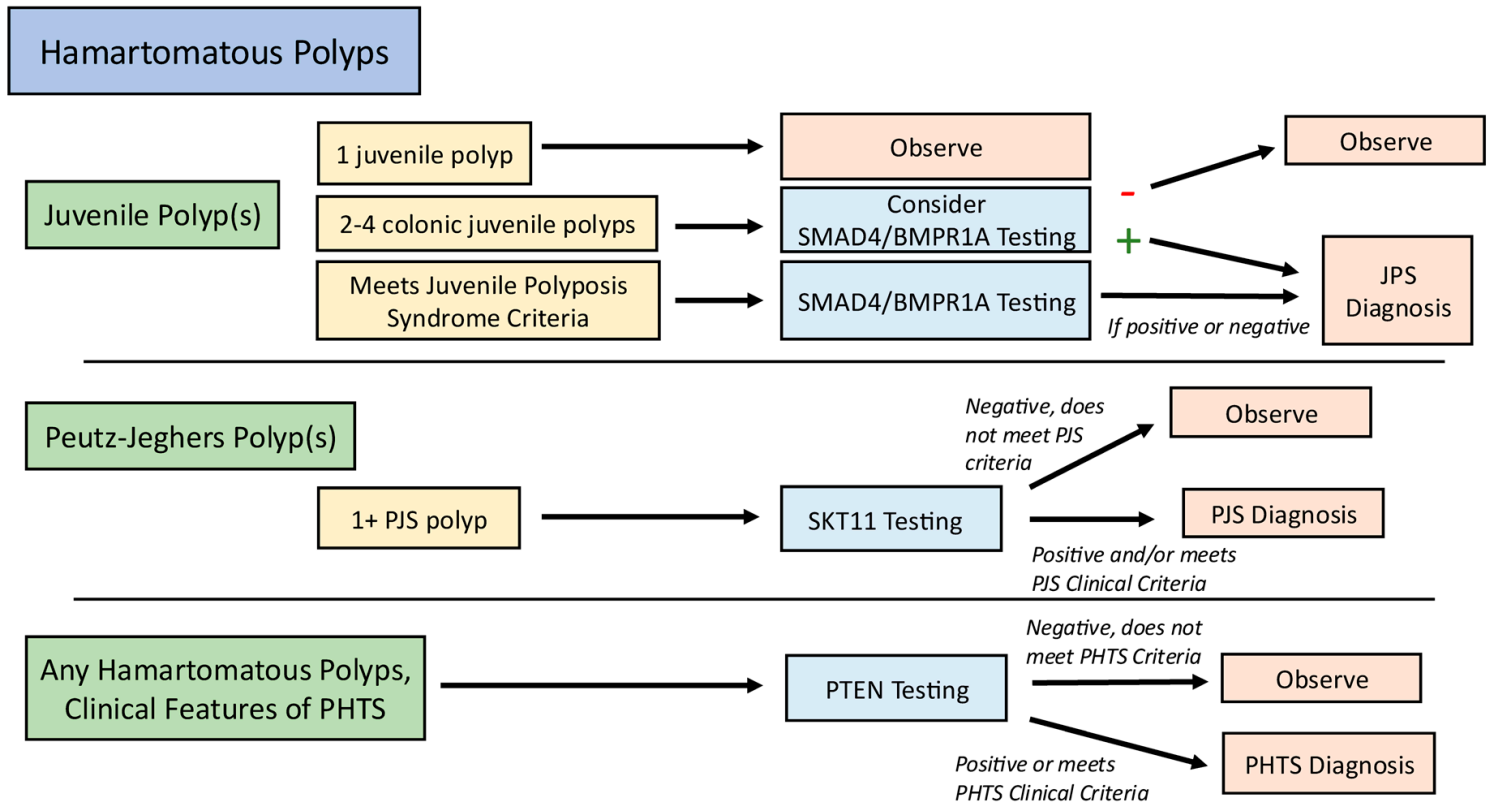
Proposed algorithm for genetic testing in a pediatric patient with hamartomatous polyps. This algorithm is based on polyp histology, established clinical and diagnostic criteria for hamartomatous polyposis syndromes, and guideline-informed recommendations for genetic testing. Where evidence-based guidelines are available, these are incorporated directly; in areas with limited pediatric data, decision points reflect expert clinical judgment. If genetic testing is negative, a patient may still meet the clinical criteria for the syndrome. Additionally, if genetic testing is negative and the syndrome is highly suspected, testing for mosaicism should be considered. *JPS:* *Juvenile Polyposis Syndrome; PHTS: PTEN Hamartoma Tumor Syndrome; PJS: Peutz-Jeghers Syndrome.*

Hamartomatous histopathology includes both juvenile polyps and Peutz-Jeghers polyps, along with inflammatory polyps and hamartomas. An approach to genetic testing for patients with hamartomatous polyps should be tailored to the specific hamartomatous polyp subtype (Figure 1).

### 3.1. Juvenile Polyps

Juvenile polyposis syndrome (JPS) is defined clinically by the presence of pathologically defined juvenile polyps in the upper and lower gastrointestinal tract. Clinical criteria are met when at least one of the following is true: (1) at least five juvenile polyps are present in the colon or rectum; (2) juvenile polyps are present in both the upper and lower GI tract; (3) at least one juvenile polyp is present and there is a family history of JPS [1]. Screening in JPS includes ongoing colonoscopy with polypectomy and pathological review for possible dysplasia [2,3]. A genetic testing approach when juvenile polyps are identified will depend upon whether clinical criteria are met and the presence of other clinical features, such as very early onset.

*Juvenile Polyp(s), Meets Clinical Criteria:* JPS is associated with germline pathogenic/likely pathogenic variants in *SMAD4* and *BMPR1A*. About half of patients with clinical JPS have a variant in one of these two genes, and fewer than half in pediatric cohorts [4,5]. There is a differential phenotype in individuals who meet the clinical criteria for JPS but do not have a causative variant (mutation-negative JPS), which leads to fewer polyps later in life and possibly a lower cancer risk [5]. Thus, individuals who do not have an identified variant will be managed differently, with the likelihood of less frequent colonoscopy in adulthood [6]. Additionally, individuals with an *SMAD4* variant should also be screened for Hereditary Hemorrhagic Telangiectasias (HHT) [2]. For this reason, *SMAD4* and *BMPR1A* sequencing and deletion/duplication analysis are recommended for patients who meet the clinical criteria for JPS.

*Juvenile Polyp(s), Does Not Meet Clinical Criteria:* If only one juvenile polyp is identified, the likelihood of identifying further polyps is approximately 17% [2,7]. For this reason, opting for clinical observation of symptoms without further endoscopic screening is a reasonable approach. If more than one juvenile polyp is identified, repeat colonoscopy in 1–2 years prior to consideration of genetic testing should be considered [7]. In either case, it is also reasonable to consider genetic testing for *SMAD4* and *BMPR1A* to prognosticate and guide further management.

*Early-Onset Juvenile Polyps:* It should be noted that an aggressive, early onset presentation of JPS raises concern for Juvenile Polyposis of Infancy (JPI), caused by deletions in 10p23 [8]. Thus, for individuals who present with multiple, early-onset juvenile polyps, especially alongside features such as macrocephaly and developmental delay suggestive of PTEN Hamartoma Tumor Syndrome (PHTS), genetic testing should be broadened to ensure coverage of this region, as it includes both the *BMPR1A* and *PTEN* genes, and microarray would be the best test if highly suspicious.

### 3.2. Peutz-Jeghers Polyp(s)

Peutz-Jeghers Syndrome (PJS) is clinically defined based on the presence of oral freckling, family history of PJS, and the presence of histologically defined PJS polyps [9]. PJS polyps pose a high risk of GI obstruction and subsequent intussusception; thus, ongoing screening for polyps and their removal is recommended, in addition to other adult-onset cancer screenings [3]. Should a PJS polyp be identified, or should a patient have other concerning clinical features, such as oral freckling, testing for *STK11* is recommended. Should a patient test negative but meet the clinical criteria for the disease, they should still undergo PJS screening, as a small percentage (<5%) of patients with PJS do not have identified *STK11* variants [10].

### 3.3. Consideration of PTEN Testing

PHTS, also known as Cowden Syndrome, is a cancer predisposition syndrome with clinical features including macrocephaly and autism spectrum disorder [11]. PHTS also predisposes to polyposis, with GI screenings recommended starting at the age of 35 years [6]. However, patients are predisposed to develop polyposis throughout their lifetime and this can develop a variety of polyp types, including juvenile polyps, GI hamartomas, ganglioneuromas, and lymphoid hyperplasia [12]. For this reason, in a pediatric patient with polyposis, especially with a mixed polyp histology, and any additional PHTS clinical features, testing for *PTEN* variants is recommended [13]. The identification of PHTS would also necessitate thyroid cancer screenings in childhood [14].

## 4. Adenomatous Polyps

**Figure 2:**
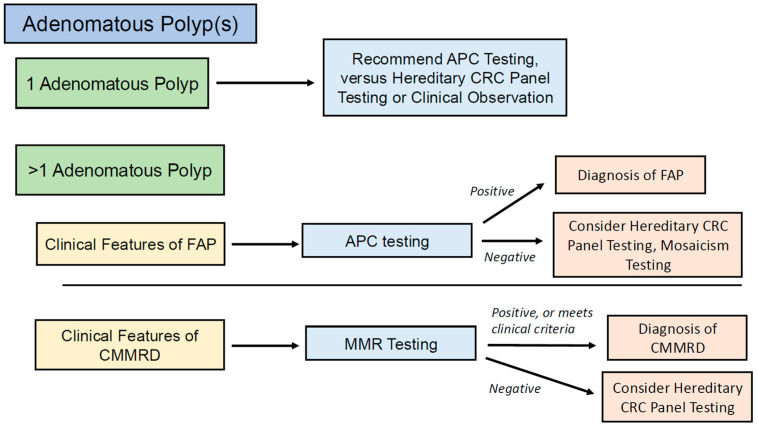
Proposed algorithm for genetic testing in a pediatric patient with adenomatous polyps. This algorithm integrates polyp burden, clinical features, and known genotype–phenotype associations to guide a stepwise approach to genetic testing. Recommendations are informed by existing guidelines where available and supplemented by expert clinical judgment in areas where pediatric-specific data are limited. If genetic testing is negative, a patient may still meet the clinical criteria for the syndrome. Additionally, should genetic testing be negative but the syndrome is highly suspected, testing for mosaicism should be considered. *CMMRD: Constitutional Mismatch Repair (MMR) deficiency; FAP: Familial Adenomatous polyposis.*

*Multiple Adenomatous Polyps:* Familial adenomatous polyposis (FAP), previously known as Gardner’s Syndrome, is an adenomatous polyposis syndrome with an up to 100% lifetime risk of colorectal cancer if left untreated [15]. Patients are also at risk for other cancer types, including upper gastrointestinal cancers, hepatoblastoma, desmoid tumors, thyroid cancer, and medulloblastoma, and thus should undergo additional systemic cancer screenings [3]. While many patients will have no clinical features of the disease apart from polyposis, some may have additional physical findings, including osteomas, supernumerary teeth, skin fibromas, epidermoid cysts, and retinal pigmented epithelium hamartomas [16]. FAP is due to germline variants in the *APC* gene, and is *de novo* in up to 25% of cases [17]. Testing for FAP is recommended in any pediatric patient with more than one adenomatous polyp (Figure 2).

Testing for Constitutional Mismatch Repair Deficiency (CMMRD) is recommended in patients with negative FAP testing with a high disease burden, or with other clinical features of CMMRD, such as a significant personal or family history of cancer, family history of Lynch Syndrome, and café au lait macules [18,19]. This would include testing for mismatch repair genes including *MSH1*, *MLH2*, *MSH6*, *EPCAM*, and *PMS2*. If testing is negative but the diagnostic criteria are still met [19], the patient should still be managed as CMMRD.

*Single Adenomatous Polyp:* A single adenomatous polyp in a pediatric patient is rare, and even without other features of FAP, testing for *APC* is recommended given the significant cancer risk associated with FAP and the rarity of adenomatous polyps in childhood [20]. If genetic testing is not done, or is done and is negative, clinical follow up with repeat endoscopic screening should be considered at a 5–10 year interval, or sooner with symptoms [21].

Should *APC* testing be negative in either scenario, testing more broadly with a polyposis panel may be recommended, as described below. Broader testing should specifically include *TP53*, especially in the presence of dysplastic polyps. Depending upon the age of the patient, a polyposis panel should also prioritize several adult-onset genes, including *POLE* and *POLD1*, both of which can cause adenomatous polyposis in heterozygous state, and *MUTYH* and *NTHL1*, both of which can cause polyposis with homozygous variants [6].

## 5. Other Rare Polyp Types

There are other, rare polyp types that uncommonly present in the pediatric age group and may require specific testing considerations. These include:

*Serrated Polyps:* Serrated polyps, sometimes also called hyperplastic polyps, are present either in isolation or associated with serrated polyposis syndrome (SPS), which is defined by the number and type of serrated lesions found in the colon and rectum [22]. The majority of patients with SPS have no identifiable genetic cause, but up to 9% have underlying germline variants with associated cancer risk, including *RNF43*, bilallelic *MUTYH* variants causing MUTYH-Associated Polyposis (MAP), *CHEK2*, and *POLD1* [23].

*Ganglioneuromas:* Ganglioneuromatous polyps are most commonly identified incidentally, but can be associated with underlying genetic syndromes [24]. Ganglioneuromas can be identified in patients with JPS and PHTS, but can also be found in individuals with other cancer risk syndromes. Diffuse intestinal ganglioneuromatosis has been reported in Neurofibromatosis Type 1 and Multiple Endocrine Neoplasia Type 2B, but isolated ganglioneuromas have not been indicative of these diagnoses [25]. For this reason, if identified in the context of a known polyposis syndrome, such as JPS or PHTS, no further genetic evaluation should be required, and if not, *BMPR1A*, *SMAD4* and *PTEN* testing should be sent.

*Mixed Polyposis:* In a pediatric patient, the identification of mixed polyp types should trigger testing for PHTS, as above, but in a case with negative *PTEN* genetic testing, there are other adult-onset cancer risk genes to consider, including *GREM1,* which is associated with Hereditary Mixed Polyposis Syndrome [26], and *NTHL1*, as biallelic pathogenic variants can also cause a mixed polyposis [27].

For these rare polyp types, although the specific genes noted here could be individually tested, a broader hereditary colorectal cancer panel would be likely to contain sufficient coverage.

## 6. Consideration of Broader Testing

Multi-gene panel testing should be considered in the case of a severe or unexpected phenotype in a patient or family member. This would include mixed polyp types with negative testing for PHTS, or polyp types not commonly seen in pediatrics, such as serrated polyps and ganglioneuromas. Similarly, when considering conditions which have a pediatric onset when caused by biallelic variants but adult onset when homozygous, this may necessitate wider panel testing. This would include, for example, Constitutional Mismatch Repair Deficiency, caused by biallelic variants in mismatch repair genes, causative of Lynch syndrome in their heterozygous state.

When sending a panel including adult-onset cancer risk genes, the age and maturity level of the patient should be considered, as it is not generally recommended to test minors for adult-onset conditions [28]. For this reason and many others, appropriate pretest counseling is crucial to ensure informed consent. Conducting genetic testing without appropriate pretest counseling has been tied to both unnecessary testing and unfavorable psychosocial outcomes [29]. Informed consent should include the pediatric patient at an appropriate developmental level to ensure patient autonomy and assent. If clinical management will not change prior to adulthood based on the genetic finding, it likely makes sense to wait until the child has reached the age of majority. Additionally, if other family members might be eligible for testing, such as a parent or grandparent with a similar polyposis history, they could be the first to undergo testing.

There are many hereditary GI cancer panel options available, and none that would be specifically recommended. It is important to remain as targeted as possible, with a preference for a smaller, more limited panel when polyposis is the only presenting feature. For example, most hereditary GI cancer panels include *CDH1*, and patholgenic variants in this gene cause Hereditary Diffuse Gastric Cancer. As screening is not effective for the detection of malignancy in this condition, gastrectomy is recommended; thus, the identification of a *CDH1* variant can be life-altering in a patient who may have no clinical symptoms of disease [6]. Similarly, the identification of a *CHEK2* variant, unlikely to be related to polyposis, can lead to stress around adult-onset cancers that will not require screening for many years, while not changing the management of the patient’s existing polyposis. For this reason, some custom panels allow for tailoring to include only the genes of interest. Alternately, whole-exome and whole-genome sequencing (WES/WGS) and/or chromosomal microarray might be considered if the patient has other phenotypic features unrelated to polyposis, such as comorbidities or syndromic physical features. This should be done in consultation with a genetics team and with appropriate phenotypic annotation to ensure polyposis genes are specifically analyzed.

In addition to the broader testing of polyposis risk genes, mosaicism should be considered as a possibility for patients with concerning clinical features but negative genetic testing. Mosaicism is an increasingly recognized cause of polyposis in patients with polyposis but negative genetic testing [30]. The cutoff for variant allele frequency can be discussed with the lab to uncover low-level variants, and the testing of polyps and normal colonic tissue can also be considered if concern for mosaicism is high.

## 7. Conclusions

A targeted genetic testing approach based on polyp pathology and clinical features is key in pediatric gastrointestinal polyposis. Given the relative scarcity of pediatric-specific data in this area, some recommendations are necessarily informed by expert opinion and extrapolation from adult studies, highlighting the need for further research to refine genetic testing strategies in children with gastrointestinal polyps. As genetic testing evolves over time, it is important to understand that clinical evaluation should remain the most important factor in the clinical follow-up of GI polyps. Additionally, the engagement of a genetic counselor and a multidisciplinary polyposis team, if available, is crucial to ensure appropriate genetic testing in pediatric polyposis.

## Data Availability

No new data were created or analyzed in this study.

## Abbreviations

The following abbreviations are used in this manuscript:

CMMRD	Constitutional Mismatch Repair Deficiency
FAP	Familial Adenomatous Polyposis
GI	Gastrointestinal
JPS	Juvenile Polyposis Syndrome
MAP	MUTYH Associated Polyposis
PHTS	PTEN Hamartoma Tumor Syndrome
PJS	Peutz-Jeghers Syndrome
